# What do hospital decision-makers in Ontario, Canada, have to say about the fairness of priority setting in their institutions?

**DOI:** 10.1186/1472-6963-5-8

**Published:** 2005-01-21

**Authors:** David Reeleder, Douglas K Martin, Christian Keresztes, Peter A Singer

**Affiliations:** 1 Department of Health Policy, Management and Evaluation, University of Toronto, Toronto, Canada; 2 University of Toronto Joint Centre for Bioethics, University of Toronto, Toronto, Canada; 3 Centre for Health Services and Policy Research, Queen's University, Kingston, Canada; 4 Department of Medicine, University of Toronto, Toronto, Canada

## Abstract

**Background:**

Priority setting, also known as rationing or resource allocation, occurs at all levels of every health care system. Daniels and Sabin have proposed a framework for priority setting in health care institutions called 'accountability for reasonableness', which links priority setting to theories of democratic deliberation. Fairness is a key goal of priority setting. According to 'accountability for reasonableness', health care institutions engaged in priority setting have a claim to fairness if they satisfy four conditions of relevance, publicity, appeals/revision, and enforcement. This is the first study which has surveyed the views of hospital decision makers throughout an entire health system about the fairness of priority setting in their institutions. The purpose of this study is to elicit hospital decision-makers' self-report of the fairness of priority setting in their hospitals using an explicit conceptual framework, 'accountability for reasonableness'.

**Methods:**

160 Ontario hospital Chief Executive Officers, or their designates, were asked to complete a survey questionnaire concerning priority setting in their publicly funded institutions. Eight-six Ontario hospitals completed this survey, for a response rate of 54%. Six close-ended rating scale questions (e.g. Overall, how fair is priority setting at your hospital?), and 3 open-ended questions (e.g. What do you see as the goal(s) of priority setting in your hospital?) were used.

**Results:**

Overall, 60.7% of respondents indicated their hospitals' priority setting was fair. With respect to the 'accountability for reasonableness' conditions, respondents indicated their hospitals performed best for the relevance (75.0%) condition, followed by appeals/revision (56.6%), publicity (56.0%), and enforcement (39.5%).

**Conclusions:**

For the first time hospital Chief Executive Officers within an entire health system were surveyed about the fairness of priority setting practices in their institutions using the conceptual framework 'accountability for reasonableness'. Although many hospital CEOs felt that their priority setting was fair, ample room for improvement was noted, especially for the enforcement condition.

## Background

Priority setting, also known as rationing or resource allocation, occurs at all levels of every health care system, including governments, funded provincial/territorial agencies, pharmaceutical benefit-management organizations, hospitals and clinical programs [1]. Countries with very different health care systems and levels of health care are all grappling with the problem of how to reconcile growing demands and constrained resources [2]. Hospitals, in particular, are struggling to meet growing demands, affordably, without compromising delivery of services [3,4].

Daniels and Sabin have proposed a framework for priority setting in health care institutions called 'accountability for reasonableness' [5-7], which links priority setting to theories of democratic deliberation, operationalizing the ethical concept of fairness. Fairness is a key goal of priority setting. According to 'accountability for reasonableness', health care institutions engaged in priority setting have a claim to fairness if they satisfy four conditions of relevance, publicity, appeals/revision, and enforcement (described in Table 1).

**Table 1:** The four conditions of 'accountability for reasonableness'

Relevance	Rationales rest on evidence, reasons, and principles that fair-minded parties can agree are relevant to deciding how to meet the diverse needs of a covered population under necessary resource constraints.
Publicity	Limit-setting decisions and their rationales must be publicly accessible.
Appeals/Revision	There is a mechanism for challenge and dispute resolution regarding limit-setting decisions, including the opportunity for revising decisions in light of further evidence or arguments.
Enforcement	There is either a voluntary or public regulation of the process to ensure that the first three conditions are met.

'Accountability for reasonableness' may be an effective framework for describing the components of priority setting, evaluating the fairness of priority setting in hospitals and identifying 'good' practices and opportunities for improvement. It can help hospitals improve their priority setting practices and can be an effective driver of health care reforms [8-10].

'Accountability for reasonableness' has been used to evaluate priority setting in health systems [11]. However, only a limited number of studies have addressed how priority setting occurs in hospitals [12-17], and no study has attempted to survey the views of hospital decision makers throughout an entire health system about the fairness of priority setting in their institutions.

The purpose of this study is to elicit hospital decision-makers' self-report of the fairness of priority setting in their hospitals using an explicit conceptual framework, 'accountability for reasonableness'.

## Methods

### Design

We conducted a survey of Chief Executive Officers, or their designates, of Ontario hospitals. The survey questionnaire covered 102 items, including hospital profile information (e.g. hospital name, number of beds, operating budgets). In this paper we focus on the results of 9 questions concerning priority setting, fairness, and the four conditions of 'accountability for reasonableness' (refer to Table 2).

**Table 2:** Survey Questions – Rating* and Open Scale

Overall, how fair is priority setting at your hospital? (Rating Scale). Please explain your response (Open-ended).
How well does your hospital meet its priority setting goal(s)? (Rating Scale). Please explain your response (Open-ended).
What do you see as the goal(s) of priority setting in your hospital? (Open-ended)
How well is the relevance condition met at your hospital? (Rating Scale)
How well is the publicity condition met at your hospital? (Rating Scale)
How well is the appeals condition met at your hospital? (Rating Scale)
How well is the enforcement condition met at your hospital? (Rating Scale)

* Ratings were on a five-point scale, from 1 (not well) to 5 (very well) or from 1 (not fair) to 5 (very fair).

### Setting and sample scope

With 12 million people, Ontario is the largest province in Canada. Like the rest of the country, it is a single payor, predominately publicly-funded health care system with some privately funded services and products (e.g. dental services, drugs). There are 160 hospitals in Ontario and all were invited to participate in this study.

### Participants

160 Ontario hospital Chief Executive Officers, or their designates, were asked to participate. 86 Ontario hospitals completed this survey, for a response rate of 54%. The average bed count of the responding hospitals was 250.4, with a range of 18 to 1265 beds. The average operating budget of the hospital sample was $75.8 million, ranging from $3.3 million to $733 million. The sampled hospitals represented a blend of teaching, small, community and specialized service facilities across the province.

### Data collection

The survey was pre-tested and mailed to Chief Executive Officers of all Ontario hospitals in January 2001. Data from returned surveys were entered into an electronic database for further analysis. Of the 9 questions analyzed in this study, 6 asked for a rating on a 5-point scale ranging from 'Not Well' or '1' to 'Very Well' or '5', and 3 were open-ended.

### Data analysis

Close-ended ratings were analyzed statistically [18], with ratings equal to and below the mid-point combined to identify proportions of respondents suggesting improvement, using a conservative bivariate cut-off point to discriminate among reported responses. P values for all hypothesis tests were two tailed. Open-ended responses were analyzed using a modified thematic analysis involving open and axial coding techniques [19,20]. In open coding, data was segmented and coded with a label identifying parts of text relating to a concept or idea. For axial coding, concepts were organized into overarching themes, and compared, both within and between questions, in search of patterns in responses and to ensure consistency.

## Results

Overall, 60.7% of respondents indicated their hospitals' priority setting was fair, while 79% stated their hospitals met their priority setting goals. With respect to the 'accountability for reasonableness' conditions, respondents indicated their hospitals performed best for the relevance (75.0%) condition, followed by appeals/revision (56.6%), publicity (56.0%), and enforcement (39.5%). (Refer to Table 3).

**Table 3:** Summary of Decision Maker Responses ^a,b^

	How well does your hospital meet its priority setting goal(s)?	Overall, how fair is priority setting at your hospital?	How well is the relevance condition met at your hospital?	How well is the publicity condition met at your hospital?	How well is the appeals condition met at your hospital?	How well is the enforcement condition met at your hospital?
# Respondents	81	84	84	84	83	81
						
5 'Very Well'	15 (18.5%)	15 (17.9%)	21 (25.0%)	14 (16.7%)	12 (14.4%)	9 (11.1%)
4	49 (60.5%)	36 (42.8%)	42 (50.0%)	33 (39.3%)	35 (42.2%)	23 (28.4%)
3	12 (14.8%)	28 (33.3%)	17 (20.2%)	26 (30.9%)	23 (27.7%)	34 (41.9%)
2	3 (3.7%)	4 (4.8%)	3 (3.6%)	9 (10.7%)	10 (12.0%)	9 (11.1%)
1 'Not Well'	2 (2.5%)	1 (1.2%)	1 (1.2%)	2 (2.4%)	3 (3.6%)	6 (7.4%)
						
(SD)	3.89 (0.84)	3.71 (0.86)	3.94 (0.84)	3.57 (0.97)	3.52 (1.00)	3.25 (1.04)

a Due to rounding, frequencies may not add up to 100%

b Based on survey scale from 1 to 5

Respondents rated the relevance ( = 3.94) condition significantly higher than the publicity ( = 3.57), appeals ( = 3.52), and enforcement ( = 3.25) conditions (p < .05) (paired-samples t test). While publicity and appeals conditions were not significantly different, respondents rated these conditions significantly higher than the enforcement condition (p < .05). The distribution of ratings suggested that there was ample opportunity for improvement, with most room for improvement in meeting the enforcement condition.

Priority setting and fairness ratings were positively correlated (r = .51, p < .01). Fairness ratings were also positively correlated with each of the 4 'accountability for reasonableness' conditions (p < .01), as were meeting priority setting goals (p < .05).

The bivariate correlation between each of the accountability variables of relevance, publicity, appeals/revision and enforcement was strongly positive (p < .01). The Pearson correlation coefficients among the 4 conditions ranged from 0.41 to 0.57. The internal consistency (Cronbach's α) of all of the 'accountability for reasonableness' conditions was very good (α = .78).

Bed size (r = -.24, p <.05) and operating budget (r = -.32, p < .01) were negatively correlated with the rating of fairness in priority setting.

### Analysis of respondent comments

In this section we describe participants' responses to the open-ended questions organized according to themes. We have included verbatim responses to help illustrate the themes.

### What do you see as the goals of priority setting in your hospital?

Decision makers emphasized four priority setting goals which were complex, interdependent, and required balancing. Some respondents said that (1) meeting needs existed in relation to delivering (2) quality services, or was constrained by resource availability. Examples of these goals include "quality care within available resources" and "access to high quality service in areas of greatest need". While (3) meeting budgets was identified as a goal by some decision makers, it did not exist in isolation from other goals such as meeting strategic directions or needs. One decision maker said his goal was "ensuring a balanced situation at year end while meeting the direction set out in our strategic plan". Achieving (4) organizational goals was expressed as a limiting, or organizational focusing process, which involved alignment or balancing of multiple goals and values.

### How well does your hospital meet its priority setting goals? Please explain your response

Decision makers described a deliberative process by which their priority setting goals were operationalized, providing examples of good practices and challenges or barriers for achievement. Overall, decision makers appeared confident that their priority setting goals were the correct ones, in no case did they comment that expressed goals were unrealistic, so contributing to lack of achievement.

Decision makers pointed to five factors of importance in meeting their priority setting goals. Review processes (1) were described in which different review processes are brought to bear pointing to areas requiring additional resources. For example, a decision maker said "annually we review and ensure that resources are being allocated to priority programs and services". Leadership (2) was implicated as an important factor in meeting priority-setting goals. For example, a decision maker said "We do not set goals that are pie in the sky, they must be achievable". With respect to (3) stakeholder consultation, some respondents pointed to the need to involve the wider community in decision making. Decision-makers felt that improvements in priority setting were contingent upon (4) access to relevant information. Finally, some decision-makers emphasized the importance of (5) decision making tools, or benchmarking, to improve decision making.

### Overall, how fair is priority setting at your hospital? Please explain your response

Respondents gave information to support their self-evaluation, and provided examples to demonstrate the fairness of priority setting processes from their perspective.

In evaluating the fairness of priority setting, decision makers emphasized five themes. Stakeholder consultation raises the level of (1) inclusivity, bringing different points of views and interests to bear in solving common problems. For example, a decision maker said "we have a service providers network in our community that has direct input into resource allocation and program planning". Respondents described review processes (2) involving the deliberation of concerned parties, using relevant data, based on need, cost and other values (e.g. evidence). Reporting systems (3) provide an institutional feedback loop, pointing to areas where limits may be fairly set. With respect to (4) revision/appeals, revising arguments or values based on iterative processes involving various stakeholders is a feature of fairness, and helps to improve buy-in, but may increase organizational tensions. Finally, (5) governance may also be involved in developing decision-making models, and in ensuring the conditions of relevancy, appeals/revision, and publicity are met. For example, a decision maker said "The Board and senior management have developed decision-making models for key program/service changes...our appeals process is informal, but open to access including the Board".

## Discussion

To our knowledge, this is the first published survey of hospital decision makers covering an entire health system, using Daniels and Sabin's framework of 'accountability for reasonableness'. It provides data to understand how fair priority setting processes are, what decision makers' priority setting goals are, and how well these goals are met.

Several studies, using either survey or qualitative methodologies sometimes in combination with hypothetical "tradeoffs", have examined priority setting from theoretical perspectives aimed at capturing the public or professional's preferences or values in priority setting [21-24]. Relatively few studies have described what is happening in real-world contexts with a view of evaluating and improving priority setting activities [25-31]. These have described actual priority setting in individual hospitals, focusing on strategic planning [12], surgery [13], critical care [14], new technologies [15], and the rationing of new drugs [17], with no study reviewing priority setting in hospitals across the health system.

This study makes five contributions to knowledge on priority setting. First, there is ample room for improvement in fair practices within Ontario hospitals as described by their Chief Executive Officers. Although many hospital CEOs felt that their priority setting was fair, ample room for improvement was noted, especially for the enforcement condition, followed by publicity, appeals/revision and relevance. While 79% of decision makers felt their hospitals met their priority setting goals, only 60.7% felt their processes were fair. According to decision makers, the perceived 'gap' was greater in meeting fairness than in priority setting goals.

Second, it is feasible for decision makers to assess the fairness of priority setting in their institutions on a quantitative basis according to the 'accountability for reasonableness' framework, with greater than half of respondents saying their processes are fair. There is a high degree of association between 'fairness' and the internal components of 'accountability for reasonableness', lending support that fairness can be operationalized through 'accountability for reasonableness'. As well, the internal conditions of 'accountability for reasonableness' are highly related, and positively correlated, suggesting relevance, publicity, appeals/revision, and enforcement are measuring comparable aspects of fairness. Consistent with this, the high Cronbach's α finding is promising for the development of an 'accountability for reasonableness' scale of perceived fairness of priority setting in health care institutions. Measuring decision makers' self-report of their perception in meeting the various conditions of 'accountability for reasonableness', the scale would provide a practical tool for decision makers to assess self-reported views over time, noting progress in meeting conditions, while providing a reference point for needed improvement (i.e. enhance meeting 'publicity' condition). Finally, the finding of a negative relationship between number of hospital beds or budgets and self-report on fairness is consistent with the view that smaller hospitals may be perceived to have fairer processes, with greater involvement of their local communities in decision making and emphasis on transparency and "trust".

Third, decision makers' views were surveyed at a health system level to determine what their actual priority setting practices were. Four complex, interrelated priority setting goals were described, suggesting a balance of need, quality, budget and organizational goals in priority determination. In addition, the study points to a close alignment of factors required in the meeting of priority setting goals and in the elements of fairness. In providing decision maker perspectives on characteristics of fairness, this study also adds to previous empirical work suggesting fair priority setting depends on a fair priority setting process [31]. Additional research is required to understand the capacity of organizations to improve such practices.

Four, this study has shown, from a blend of qualitative and quantitative approaches, that it is feasible to operationalize the 'accountability for reasonableness' framework, through the design of survey instruments to facilitate self-evaluation, and identification of good practices. This data can be shared with decisions makers to improve the fairness of their priority setting processes in meeting the four conditions of 'accountability for reasonableness'.

Finally, this study expands on the likely relationship between leadership and priority setting in which leadership was found to contribute to perceptions of fairness in two committees engaged in priority setting for new health care technologies. Study results indicate that greatest room for improvement exists in meeting the 'enforcement' condition, with decision-makers describing critical leadership success factors, including: the role of governance in establishing policy and in meeting this condition, the need to set achievable corporate goals, and the significance of a lobbying funding function. Further study is needed to clarify the nature of this leadership contribution.

### Limitations

The study is limited, first, in the response rate from hospitals. Only 54% of Ontario's 160 hospital CEO's responded to the survey. It is possible that hospitals responding to this survey were those doing, or perceived doing, better on self-report than others. However, there is no evidence to suggest that this is the case, and the sample did include small, medium and larger teaching hospitals, as well as specialized facilities to mitigate against representative selection bias. Second, social desirability bias was possible in that the views of decision makers expressed in the survey may not have corresponded to what they actually believed, or did. However, unpublished data [32] based on in-depth interviews with Ontario hospital decision makers suggest self-reported views in relation to fairness were similar in both survey respondents and non-respondents, and that reasons for non-response were related to convenience, workload and priority, and not social desirability. Third, corroborative evidence of 'fairness', obtained through in-depth interviews with staff or community stakeholders, or through review of other relevant information (e.g. meeting minutes, publication of rationales on web sites) was not done. The study design was a survey, focusing on the self-report of fairness from the perspective of Chief Executive Officers, or delegates. In-depth interviews with Chief Executive Officers, or additional individual hospital case studies would be helpful in understanding actual priority setting practices, building on these survey results.

## Conclusions

In this first survey of Chief Executive Officers within a health region reporting on their assessment of the fairness of priority setting practices in their institutions according to 'accountability for reasonableness', ample room for improvement was observed, especially for the enforcement condition. Additional study is required to understand how application of 'accountability for reasonableness' will vary within and across institutions, and is shaped or influenced by various parameters, including the nature of corporate leadership. These evaluations can help to identify good practice opportunities for improvement that can be shared between local institutions or used within government to drive health care reforms.

## Competing interests

The author(s) declare they have no competing interests.

## Authors' contributions

DR was the primary analyst and principal author of the manuscript. DKM, CK and PAS were involved in study conception, analysis and drafting the manuscript. CK and DKM were involved in data collection. All authors read and approved the final manuscript.

## Pre-publication history

The pre-publication history for this paper can be accessed here:


